# Linking administrative data on children's social care to investigate the outcomes of provision

**DOI:** 10.23889/ijpds.v8i2.2254

**Published:** 2023-09-14

**Authors:** Rick Hood, Allie Goldacre

**Affiliations:** 1 Kingston University, Kingston upon Thames, United Kingdom

## Objectives

This paper will present findings from a study of linked administrative data on children’s social care (CSC) services in England. Its objectives were to identify and profile the underlying categories of demand for CSC services in England and compare the outcomes of provision between these categories, using a range of measures.

## Method

An anonymised longitudinal dataset was assembled for children in need and children in care who received services in England between 2015-21. Latent Class Analysis (LCA) was used to identify mutually exclusive categories of demand for all children subject to a statutory social work assessment within that period, based on the factors identified at assessment. Descriptive profiles of these categories were constructed using demographic data as well as children’s intervention pathway following assessment. Survival analysis using adjusted Cox regression models was then undertaken to compare rates of re-referrals, repeat child protection plans, and re-entries to care, for children receiving services within each category.

## Results

The latent class analysis found twelve distinct categories of demand for children’s social care services, which were consistent across local authorities. Conditional probabilities were used to interpret the demand represented by each category, in consultation with stakeholders. The most prevalent category was domestic abuse and violence (19.7%), followed by complexities around parental mental health (18.4%). Other categories included disability, child mental health, risks outside the home, complex domestic abuse, and concerns about another person in the family or household. The profile of children varied across these categories, including age, gender, ethnicity, the level of risk identified by services, and the type of provision received. Significant differences were also found in the outcomes of provision across categories, especially in re-referrals and re-entries to care.

## Conclusion

The categories create an evidence-based typology of demand to supplement conventional performance measures. Local profiling of these categories can be support designing and planning services and aligning the textual information gathered in assessments with the statistical data used for audit and inspection.

